# Nrf2与铁死亡在肿瘤耐药中的研究进展

**DOI:** 10.3779/j.issn.1009-3419.2023.101.31

**Published:** 2023-10-20

**Authors:** Shuning HU, Xinru ZOU, Yixuan FANG, Chengrui LIU, Rui CHEN, Lili JI

**Affiliations:** ^1^226001 南通，南通大学医学院病理解剖学系; ^1^Department of Pathological Anatomy, School of Medicine, Nantong University; ^2^226001 南通，南通大学附属医院病理科; ^2^Department of Pathology, Affiliated Hospital of Nantong University, Nantong 226001, China; ^3^215006 苏州，苏州大学附属第一医院病理科; ^3^Department of Pathology, First Affiliated Hospital of Soochow University, Suzhou 215006, China

**Keywords:** 肿瘤, 耐药, 铁死亡, Nrf2, Cancer, Drug resistance, Ferroptosis, Nrf2

## Abstract

肺癌作为全球最常见的癌症之一，其治疗策略主要为手术结合放化疗。但是，长期化疗会产生耐药，也是肺癌治疗的难点之一。铁死亡是当细胞中存在氧化还原稳态失衡时，一种铁依赖和脂质过氧化驱动的非凋亡性细胞死亡级联反应。细胞核因子E2相关因子2（nuclear factor erythroid 2-related factor 2, Nrf2）是细胞抗氧化反应的关键。大量研究表明Nrf2信号通路在铁、脂质和氨基酸代谢等方面具有多种功能，在铁死亡调控中发挥重要作用。因此，本文对近十年来铁死亡的研究进展作一简要综述。特别是，将讨论铁死亡的机制和Nrf2对铁死亡的调节，以及Nrf2通路与铁死亡在肿瘤耐药中的作用，为耐药肺癌患者的治疗提供新的研究方向。

## Abstract (en)

Lung cancer is one of the most common cancers in the world, and its treatment strategy is mainly surgery combined with radiotherapy and chemotherapy. However, long-term chemotherapy will result in drug resistance, which is also one of the difficulties in the treatment of lung cancer. Ferroptosis is an iron-dependent and lipid peroxidation-driven non-apoptotic cell death cascade, occurring when there is an imbalance of redox homeostasis in the cell. Nuclear factor erythroid 2-related factor 2 (Nrf2) is key for cellular antioxidant responses. Numerous studies suggest that Nrf2 assumes an extremely important role in regulation of ferroptosis, for its various functions in iron, lipid, and amino acid metabolism, and so on. In this review, a brief overview of the research progress of ferroptosis over the past decade will be presented. In particular, the mechanism of ferroptosis and the regulation of ferroptosis by Nrf2 will be discussed, as well as the role of the Nrf2 pathway and ferroptosis in tumor drug resistance, which will provide new research directions for the treatment of drug-resistant lung cancer patients.

肺癌是最常见的癌症之一，据估计，截至2020年，每年有220万新病例和179万人死亡^[1]^。早期肺癌多以手术治疗为主，而晚期肺癌患者的一线治疗方案是以顺铂为基础的联合化疗，但长期化疗会产生耐药，也是肺癌治疗的难点之一。

细胞核因子E2相关因子2（nuclear factor erythroid 2-related factor 2, Nrf2）最早发现参与氧化应激的适应性调节因子^[2]^。Nrf2的信号通路主要受Kelch样环氧氯丙烷相关蛋白1（Kelch-like ECH associated protein 1, Keap1）负性调控，后者在非应激条件下，通过泛素化来诱导Nrf2的蛋白酶体降解^[3]^。在氧化应激中，抗氧化因子Nrf2被激活以保护细胞免受氧化应激损伤，从而导致肿瘤细胞化疗耐药，然而其具体机制仍然有待研究。研究^[4]^表明，Nrf2通路在肺癌中常处于异常活化状态，在人类多种恶性肿瘤中，Keap1和Nrf2的基因改变频率最高的为肺癌。肺癌中高组成型Nrf2活性可以引发肺癌恶性进展和对治疗的药物抵抗。

肿瘤耐药是导致患者治疗失败的主要原因，其发生涉及体内多通路及基因激活表达。肿瘤细胞耐药程度与细胞内活性氧（reactive oxygen species, ROS）刺激引起的氧化应激防御通路激活有着密不可分的联系。ROS对细胞生命至关重要，它们在细胞外信号的转导和控制中扮演着第二信使的角色，并调控着细胞增殖、分化和存活相关基因的表达。事实上，ROS在癌症中具有双重重要性。一方面，它们可以促进正常到癌细胞的修饰发生；另一方面，持续的氧化应激使癌细胞对额外的应激源越来越脆弱，并逆转肿瘤耐药性。铁死亡是一种非凋亡形式的细胞死亡^[5]^，其发生的主要原因和特征是细胞的抗氧化能力下降，导致细胞内出现铁超载和ROS依赖的脂质过氧化物累积^[6]^。并且铁死亡与癌症治疗的耐药性相关，诱导铁死亡可以逆转肿瘤耐药性^[7]^。过多的ROS可导致氧化应激和严重后果。然而，身体可以使用抗氧化防御机制来维持ROS平衡。而作为氧化应激信号的主要调节者，Nrf2可通过调控其靶基因的表达来影响铁死亡。

本文将从Nrf2的结构、调控与功能，铁死亡的发生机制，Nrf2在抗脂质过氧化和铁死亡中的作用，靶向Nrf2和铁死亡作为克服肿瘤耐药的癌症疗法等4个方面进行综述，为耐药肺癌患者的治疗提供新思路。

## 1 Nrf2的结构、调控与功能

### 1.1 Nrf2的结构

肺癌是全球癌症中死亡率最高的癌症，大多数死亡与转移有关，其中1/3的肺癌有Nrf2突变^[8]^。由基因NFE2L2编码的转录因子Nrf2，含有一高度保守的碱性亮氨酸拉链（basic leucine zipper, BZIP）结构，并被鉴定为帽领（Cap-n-collar, Cnc）家族的成员，包括核因子红系衍生2（nuclear factor-erythroid 2, NFE2）和Nrf1、Nrf2、Nrf3^[9]^。而由于在氧化应激反应中的细胞保护作用，Nrf2是其中最著名的家族成员^[10]^。通过细胞氧化还原稳态，Nrf2可改变细胞对外源性物质和氧化应激的适应性反应来保护细胞。从Neh1到Neh7，Nrf2的蛋白质结构由7个结构域组成^[2]^。为了与Maf蛋白和DNA相互作用，Neh1结构域具有CNC-bZIP区域。DLG和ETGE是Neh2的N端上的两个结合位点，它们募集以结合Kelch，与ECH相关蛋白1（Keap 1）相似，后者是Nrf2的负性调控分子。在Neh3的C-末端基于cullin E3泛素连接酶（Cul3）的A区将通过募集解旋酶DNA结合蛋白和染色体ATP酶参与上游基因传递（CHD）。Neh4和Neh5区吸引SRC3和cAMP元件结合蛋白（cAMP-response element binding protein, CREB）与靶基因的结合。Neh6区域中的DSGIS和DSAPGS基序与含有β转导重复序列的蛋白（beta-transducin repeats-containing proteins, β-TrCP）相关，通过募集Rocl核心E3泛素连接酶来抑制Nrf2，Neh6与Nrf2氧还非依赖的负性调节有关。最后一个结构域Neh7区域可以通过与类视黄醇X受体结合来减少抑制转录^[11]^。

### 1.2 Nrf2通路的调控

Nrf2及其下游靶基因的激活是一个复杂的过程，研究其激活机制是当前的重点。

#### 1.2.1 Keap1介导的Nrf2降解机制

Keap1作为氧化应激敏感性的分子开关，可负性调控细胞内Nrf2的蛋白水平。其通过Cullin3依赖的E3泛素酶连接底物，可以捕捉Nrf2并迅速使其泛素化，然后转至26S蛋白酶体降解，从而使细胞内的Nrf2在未受到刺激时保持低水平；而在氧化应激条件下，Keap1分子构象发生改变，Nrf2从Keap1上脱离，并在细胞核中与Maf蛋白结合形成Nrf2-Maf异二聚体，此异二聚体可以识别抗氧化反应元件（antioxidant response element, ARE）基因序列，并诱导抗氧化和代谢基因的表达^[12]^。

#### 1.2.2 Nrf2磷酸化对Nrf2的降解机制

Nrf2是蛋白激酶C（protein kinase C, PKC）、酪蛋白激酶2（casein kinase 2, CK2）、酪氨酸蛋白激酶Fyn、糖原合酶激酶3（glycogen synthase kinase-3, GSK-3）等一些蛋白激酶的底物^[13]^。研究^[14]^发现，除了氧化应激及Keap1介导的Nrf2泛素化修饰直接参与Nrf2的调控，胞内Nrf2的负调节还可通过几种激酶的磷酸化而发生。例如，PKC作为激酶可介导Nrf2发生磷酸化，磷酸化的Nrf2分子得以从其负性调控分子Keap1中释放出来并入核，从而启动相关基因的表达。此外，CK2对Nrf2蛋白结构域的磷酸化是神经母细胞瘤中Nrf2发生核定位及转录激活的必须环节^[15]^。同时磷酸化的Fyn可在细胞核中积累，并使Nrf2的Y568处酪氨酸发生磷酸化，这会导致Nrf2的核输出、泛素化和降解，并导致下游靶基因转录受影响^[16]^。Nrf2在其Neh6结构域上受两个不同的β-Trcp识别基序控制，其中一个可以受到糖原合成酶激酶-3β（glycogen synthase kinase-3β, GSK-3β）活性的调节，并导致其磷酸化从而引起Keap1非依赖性的泛素化降解^[17]^；另一个机制涉及GSK-3β的活化导致核Fyn激酶的磷酸化和易位，导致Nrf2在酪氨酸568处的磷酸化，从而刺激Nrf2从核中移出并通过蛋白体的降解^[18]^。

### 1.3 Nrf2的功能

在肿瘤的发生和发展中，Nrf2作为多效性转录因子，发挥着肿瘤抑制和肿瘤促进的作用。

#### 1.3.1 Nrf2抗氧化细胞保护作用

作为重要的抗氧化转录因子，Nrf2可通过激活一系列含有ARE基因序列的靶基因来降低ROS水平，维持细胞的ROS相对稳态，这些基因负责一部分调节谷胱甘肽（glutathione, GSH）代谢，包括谷氨酸/胱氨酸反向转运蛋白xCT、谷氨酸-半胱氨酸连接酶（glutamate-cysteine ligase, GCLC/GCLM）、硫氧还蛋白（thioredoxin, TXN）和谷胱甘肽合成酶（glutathione synthetase, GSS）等；另一部分负责酶促抗氧化系统，包括含硒半胱氨酸抗氧化蛋白GPX、喹啉酮氧还蛋白1（NADPH quinone oxidoreductase 1, NQO1）等^[19]^。因此，Nrf2被普遍认为是细胞抗氧化反应的主要调节剂。在许多与氧化应激相关的疾病中，Nrf2激活的细胞保护作用已被报道^[10]^；因此，对各种天然来源（食品和药用植物）激活Nrf2的能力以及用作抗氧化剂和化学预防剂的能力进行了测试^[20]^。在健康组织中，Nrf2信号通路的瞬时激活通过调节多种不同的转录网络来阻止癌症的发生，在氧化应激过程中，超过1000个具有ARE的基因启动子可被Nrf2激活^[21]^。

#### 1.3.2 Nrf2调节控制增殖基因的基础和诱导型表达

例如Nrf2可通过调控NOTCH 1、NPNT、BMPR1A、IFG1、ITGB2、PDGFC、VEGFC和JAG1等促进肿瘤细胞的增殖；并且为了支持瘤细胞的增殖和生长，使癌细胞具有更高的蛋白质合成速率，通过ATF4的活化来调节丝氨酸/甘氨酸生物合成途径基因的表达（包括PHGDH、PSAT1、PSPH、SHMT1和SHMT2）^[22]^。

#### 1.3.3 Nrf2通路可促进肿瘤细胞抗凋亡及耐药

Nrf2的短暂激活可以保护氧化应激下的正常细胞，而癌细胞中Nrf2的过度激活可能有助于它们在氧化应激下生存和适应。这可能是有害的，与癌症进展和化疗耐药性有关。Nrf2可调控多药耐药蛋白（multidrug resistance-associated protein, MRP）家族基因，包括MRP1、MRP2、MRP3、MRP4等多个基因的转录，这些基因在细胞防御中起关键作用，因此，Nrf2通路过度激活很大可能决定了肿瘤对放化疗的抵抗性。并且Nrf2通过诱导BCL-2和BCL-xL的表达，减少细胞色素c从线粒体的释放，以及在用细胞毒性剂如顺铂或依托泊苷处理后减少胱天蛋白酶3/7活化来直接抑制细胞凋亡^[23]^。而且有研究^[24]^显示，Nrf2通过ROS非依赖的同源重组修复途径促进辐射诱导的DNA损伤修复，从而导致肿瘤细胞对放疗和化疗的耐药。因此，抑制Nrf2活性可能是使癌细胞对抗癌药物敏感的有效途径。

## 2 铁死亡的发生机制

### 2.1 铁死亡的基本定义

铁死亡最初是由Dixon等^[25]^在2012年发现的一种细胞死亡形式，最初是通过高通量筛选抗肿瘤药物发现，一开始发现小分子Erastin和RSL3能对RAS突变的肿瘤细胞诱发一种独特的铁依赖性的细胞死亡，并且它在遗传学和生物化学上不同于其他类型的细胞死亡，如细胞凋亡。随着对铁死亡的越来越深入的研究，它的定义越来越清晰，主要有3个方面：游离铁的增加，脂质过氧化物的积累以及在形态上与自噬、凋亡或坏死性细胞死亡不同的死亡类型^[26]^。在形态学方面，铁死亡导致线粒体变小，线粒体膜密度增加，细胞在收缩之后，细胞质成分凝结成“膨胀”表型，包括形成主要由空胞质溶胶组成的清晰、圆形的形态，还涉及质膜变薄、不稳定，显著的细胞骨架重排和蛋白质稳态的普遍破坏^[27]^。

### 2.2 铁死亡发生的主要条件

铁死亡的本质是GSH的耗竭，引起谷胱甘肽过氧化物酶4（glutathione peroxidase 4, GPX4）活性下降，细胞内铁代谢失衡造成不稳定铁水平增高，使脂质氧化物不能通过GPX4催化还原，之后与二价铁离子发生反应产生脂质活性氧，从而促使铁死亡的发生。铁积累、自由基产生及多不饱和脂肪酸（polyunsaturated fatty acids, PUFAs）参与的代谢紊乱对于诱导铁死亡是至关重要的。

### 2.3 铁死亡的调控机制

#### 2.3.1 脂质过氧化途径

PUFAs的氧化是值得注意的铁死亡指标。当PUFA存在于细胞膜中时，对脂质过氧化敏感，最容易产生过氧化反应，这可能导致对这些膜的有害影响。铁死亡的特征是ROS诱导的脂质过氧化为特征的细胞死亡，主要影响含有PUFA的特定磷脂并直接损害细胞膜的完整性。参与细胞膜中PUFAPE生物合成和修饰的关键酶包括ACSL4和LPCAT3。因此，在ACSL4和LPCAT3的参与下，PUFA被活化，然后与PE和PC结合形成PUFA-PE/PC复合物^[28]^。脂氧合酶（lipoxygenases, LOXS）通过促进铁死亡途径中PUFA-PE的过氧化而在促进铁凋亡中起重要作用^[29]^。下一步涉及PUFA-PE/PC通过与游离铁的芬顿反应（Fenton reaction）转化为PI-OOH。PI-OOH的产生增加可使膜不稳定，特别是在渗透压下，导致铁死亡。并且PI-OOH可引发新的PUFA氧化以产生细胞死亡诱导化合物，如4-HNE和丙二醛。因此，构成细胞膜的PUFAs的氧化是铁死亡的一个重要特征。过量的脂质过氧化将PUFA转化为PUFA PI-OOH，并分解细胞膜，使细胞发生铁死亡。

#### 2.3.2 细胞内GSH的缺乏

细胞膜转运蛋白胱氨酸/谷氨酸转运蛋白（也称为Xc-系统）以1:1的谷氨酸反向转运将胱氨酸（cystine）导入细胞。在胱氨酸/谷氨酸反向转运系统中，氨基酸不能直接扩散到细胞中，如需穿过细胞膜必须在特定运输蛋白的帮助下才能完成。细胞膜上的Xc-系统是运输工具之一，由轻链SLC7A11和重链SLC3A2两个核心部分组成。胱氨酸一旦进入细胞，可用于合成还原型GSH，而后者是细胞内重要的抗氧化剂。它在细胞内的合成分两步：首先，胱氨酸可以被氧化成半胱氨酸（cysteine, Cys），与谷氨酸由谷氨酸-Cys连接酶（glutamate-cysteine ligase, GCL）连接到一起；随后，其产物与甘氨酸在GSS催化的反应中合成GSH。通过使用GSH作为还原辅因子，GPX4能够将脂质氢过氧化物还原成脂质醇，因此GSH-GPX4抗氧化系统在保护细胞免于铁死亡中具有重要作用^[30]^。SLC7A11所属的System Xc-被抑制可导致Cys消耗，造成GSH合成底物的缺失，从而削弱抗氧化酶GPX4的功能，导致氧失衡，最终导致铁死亡。

#### 2.3.3 铁代谢相关的铁死亡调节途径

铁是参与氧化还原反应的几种酶的基本辅因子，因为它能够以两种离子形式存在：二价铁（Fe^2+^）和三价铁（Fe^3+^）。然而，铁的氧化还原能力可导致氧自由基的产生，其可破坏各种细胞组分。尤其Fe^2+^可以与过氧化氢通过芬顿反应形成羟基自由基，随后与PUFA反应形成脂质过氧化物引起膜损伤。为了防止铁中毒，游离不稳定铁（Fe^2+^）在系统和细胞水平上被多个系统控制，以维持铁稳态。过剩的游离铁作为不稳定铁库（labile iron pool, LIP）的一部分存在，LIP的变化是由摄取增加、储存减少、含铁蛋白质分解或铁输出蛋白功能障碍引起的^[31]^。细胞铁稳态伴随着铁蛋白和包括转铁蛋白在内的其他蛋白质的协调调节表达。细胞具有调节铁代谢的转铁蛋白受体（transferrin receptor, TFRC）。铁是通过Fe^3+^负载的铁蛋白的内吞作用与其TFRC在严格调控的反馈控制下相互作用而摄入的，而细胞膜蛋白SLC40A1则介导铁输出。大部分细胞内铁可以过铁蛋白以Fe^3+^形式储存。铁蛋白吞噬是用来描述通过自噬机制去除主要的铁储存蛋白即铁蛋白。在此背景下，已知3种铁储存蛋白，包括铁蛋白重链1、铁蛋白轻链和线粒体铁蛋白。核受体共激活因子4（nuclear receptor coactivator 4, NCOA4）是一种胞质自噬受体，用于结合铁蛋白，随后通过选择性自噬来释放铁蛋白中的铁离子，有助于铁死亡的发生^[32]^。并且，有研究^[33]^显示NCOA 4的遗传敲低可预防Erastin诱导的铁死亡，而单独NCOA 4的过表达足以引起铁死亡表型。因此，铁代谢对于铁死亡的调控关键在于对不稳定铁池容量的调控。通过运用相关诱导剂或抑制剂调节铁的转运与吸收，或许是调节细胞铁死亡水平的有效方法。

## 3 Nrf2在抗脂质过氧化和铁死亡中的作用

由于铁死亡是一种氧化形式的细胞死亡，Nrf2最初被认为主要通过调节抗氧化反应来发挥其抗铁死亡作用。然而，研究^[26]^揭示了Nrf2调控铁死亡的新机制，该机制超出了抗氧化功能，延伸到了铁和脂质稳态的关键层面。在这篇综述中，我们将深入探讨Nrf2如何通过调控铁稳态和脂质过氧化在介导铁死亡调节方面发挥重要作用。参与铁死亡调节的Nrf2靶基因，根据其功能，可将其分为3大类：铁代谢、中间代谢和GSH合成代谢。

研究^[34]^表明，几个Nrf2靶基因调控铁稳态的关键方面，包括血红素的生物合成和分解代谢，以及铁的吸收、输出、储存和利用。铁蛋白负责隔离游离铁并阻止其参与芬顿反应。铁蛋白笼子由铁蛋白重链和铁蛋白轻链的24个重复亚基组成，两者的启动子区域均含有ARE；并且铁运素（SLC40A1/FPN1）介导过量Fe^2+^的输出，也被鉴定为含有ARE，并可被Nrf2转录上调^[35,36]^。由于体内功能铁的重要组成部分是血红素，血红素代谢异常可增加铁死亡的风险。许多参与血红素代谢的Nrf2靶基因最初通过Chip-seq分析鉴定出来，包括ATP结合盒亚家族B成员6、亚铁螯合酶（ferrochelatase, FECH）和溶质载体家族成员48成员A1以及血红素转运蛋白都被Nrf2上调；血红素加氧酶1（heme oxygenase-1, HMOX1）是一种催化血红素降解产生胆绿素的酶，最初被鉴定为姜黄素处理的肾上皮细胞中Nrf2的靶基因^[37⇓⇓-40]^；胆绿素还原酶A和B也可通过Nrf2活化直接上调^[35]^。总的来说，Nrf2对铁稳态的调节是细胞对铁死亡敏感性或抗性的关键决定因素，而这独立于其抗氧化功能。

除铁和血红素外，Nrf2还调节参与中间代谢的代谢物，其中许多对于反应性中间体的分解代谢/解毒和还原型烟酰胺腺嘌呤二核苷酸磷酸（nicotinamide adenine dinucleotide phosphate, NADPH）（还原铁和血红素所需的关键电子供体）再生是重要的，后者（NADPH）是还原氧化底物所需的关键电子供体。脂质合成高度消耗NADPH，并与细胞抗氧化系统竞争。研究^[41]^表明，激活Nrf2可减轻小鼠肝脏中脂肪酸的生成和去饱和，从而增加NADPH含量，实现小鼠肝脏的解毒作用。核受体亚家族O,B组成员2（nuclear receptor subfamily O, group B, member 2, NROB2）和过氧化物酶体增殖物激活受体γ（peroxisome proliferation-activated receptor γ, PPARγ）是核受体，在胆固醇代谢的调节和控制中发挥重要作用，可能有助于抗过氧化作用。这两个基因都可以被Nrf2激活，从而在缓解脂质过氧化物积累中发挥关键作用^[42⇓-44]^。

此外，Nrf2在GSH的合成代谢过程中也发挥重要作用。SLC7A11是System Xc-的两个亚基之一，在GSH代谢中受到Nrf2的调控，它负责将Cys转运到细胞中，从而增加Cys含量，促进GSH的生成。在GSH生物合成过程中，还有两种重要的酶受到Nrf2正向调控，包括GCLC/GCLM和GSS^[45⇓⇓⇓-49]^。溶质连接载体家族A1成员5可被Nrf2激活，在谷氨酰胺摄取中发挥重要作用，有助于GSH的生物合成。此外，Nrf2还在转录水平上调控许多氧化还原酶，这些酶包括：谷胱甘肽-S-转移酶GSTP1和GSTA1、过氧化物氧还蛋白PRDX1和PRDX6、硫氧还蛋白还原酶TXNRD1^[50]^。最重要的是，作为对抗脂质过氧化的重要作用而参与铁死亡调控的一个关键酶，GPX4也受到了Nrf2的转录调控^[51]^。总之，通过调节Nrf2信号通路促进或抑制铁死亡的疗法是可行的，值得花费时间和精力进行探索。

## 4 靶向Nrf2和铁死亡作为克服肿瘤耐药的癌症疗法

铁死亡最初是研究者通过高通量筛选针对RAS基因转化致癌细胞的抗癌药的研发过程中发现的，研究者^[52]^发现过表达RAS癌细胞会对Erastin启动的非凋亡死亡过程敏感。研究表明，对传统抗肿瘤药物产生耐药的患者而言，诱导肿瘤细胞铁死亡可作为其新的抗癌治疗策略。近年来，人们发现了越来越多的铁死亡诱导剂，并在许多临床前癌症模型中证实了它们用于杀死耐药癌细胞的作用。随着我们对Nrf2抗铁死亡作用的了解越来越多，可以明显预测抑制Nrf2将显著增强铁死亡诱导剂的作用。此外，许多癌细胞具有组成性激活的Nrf2，这导致细胞保护基因的上调，从而促进肿瘤进展并保护癌细胞免受化疗损伤，这被称为Nrf2的黑暗面^[53]^。在不同的癌症模型中，激活Nrf2可以预防铁死亡。例如，自噬受体p62依赖的Keap1的隔离导致Nrf2上调，降低了肝细胞癌对Erastin和索拉非尼诱导的铁死亡的敏感性^[54]^。另一项使用头颈癌细胞的研究^[55]^表明，Nrf2对这些细胞逃避RSL3诱导的铁死亡反应至关重要。采用CRISPR筛选的3D培养模型揭示了高度活跃的Nrf2通过调节增殖和铁死亡而成为肺肿瘤球状体形成的先决条件^[56]^。顺铂（Cisplatin, DDP）被认为是治疗晚期非靶向肺癌的一线药物。然而，对DDP的耐药性也是肺癌治疗的一个主要障碍。有研究^[57]^表明，Isoorientin与DDP联合治疗，通过体外和体内实验，可导致耐药细胞的生存能力明显下降。在机制上，Isoorientin作为调控SIRT6/Nrf2/GPX4信号通路的调节剂（下调SIRT6蛋白、Nrf2和GPX4的表达），从而调节肺癌细胞的铁死亡，逆转耐药。因此，Nrf2的水平与铁死亡敏感性直接相关，通过调控Nrf2表达水平将影响癌细胞对铁死亡的敏感性^[54,58]^。

在寻找诱导铁死亡化合物的大筛选中，发现了一系列小分子诱导剂。大多数铁死亡诱导剂其作用机制与最初鉴定的两种铁死亡诱导化合物Erastin和RSL-3类似，即通过抑制xCT系统造成GSH耗竭或直接阻断GPX4的活性^[59]^。正如所预期的，基于Nrf2对xCT及GPX4这两个靶标以及许多其他关键途径的转录调节，抑制Nrf2可显著增强促铁死亡试剂的治疗敏感性。事实上，用胡芦巴碱作为一种Nrf2抑制剂，其治疗已经被证实是有效的，可阻断血红素和铁代谢相关的Nrf2靶基因的表达，与Erastin和索拉菲尼协同作用，可促进肝癌细胞的铁死亡^[54]^。对头颈癌的研究^[60]^发现，Nrf2通路激活有助于头颈癌细胞对青蒿琥酯的耐药性，通过使用胡芦巴碱抑制该通路可消除头颈癌细胞对青蒿琥酯诱导的铁死亡耐药。此外，零价铁纳米颗粒（ZVI-NP）因其可用于去除地表和地下废水中有毒重金属而在环境污染治理中发挥重要作用，最近因其抗肿瘤疗效，现在正逐渐进入人们的视线^[61]^。已有研究^[62]^发现ZVI-NP在癌细胞的溶酶体中而不是在正常细胞中优先转化为Fe^3+^，因为癌细胞内部更具酸性。铁离子的突然释放进一步诱导癌细胞中的ROS激增，并导致癌细胞中的铁死亡。为了探索ZVI-NP在肺癌模型中调节肿瘤微环境的潜在作用，一项研究^[63]^鉴定了其诱导铁死亡的新分子机制。从机制上讲，ZVI-NP增强了Nrf2的磷酸盐依赖性泛素化降解，从而因过度氧化应激和脂质过氧化引发铁死亡。所有这些都揭示了ZVI-NP在癌症治疗方面的潜力，从而减少了副作用并提高了疗效。总的来说，我们有理由相信，未来会有越来越多的疗效和预后更好的治疗策略出现。靶向Nrf2信号通路和诱导铁死亡的药物应用于肿瘤的治疗是一种新颖且有前景的方法。

## 5 靶向Nrf2信号通路抑制铁凋亡在其他疾病治疗中的应用

Nrf2和铁死亡之间的联系也已在癌症以外的疾病的发病机制中得到阐明，表明在其他病理学背景下靶向该级联反应的可能相关性。例如，对阿尔茨海默病和帕金森病以及心血管疾病的研究^[27,64]^表明，铁死亡可能是这些疾病的主要驱动因素。Nrf2的激活可以上调xCT的表达，增强谷氨酸分泌，并恢复GPX4活性，从而抑制铁凋亡。随着年龄的增长，Nrf2活性的逐渐丧失被认为增加了对铁死亡的易感性，最近已经证明铁死亡在阿尔茨海默病、帕金森病、多发性硬化和其他神经退行性疾病的发病机制中起关键作用^[27]^。因此，激活Nrf2以抑制铁死亡可能成为治疗神经退行性疾病和心血管疾病的新途径。DMF（Tecfidera®）可视为激活Nrf2的最成功病例^[65]^。该药物最初用于治疗银屑病患者，但新的研究^[66]^表明，在进入体内后，它发生了转化，从而共价修饰KEAP1上的关键Cys残基，从而阻断Nrf2泛素化并促进Nrf2稳定以及Nrf2靶基因的后续激活。此外，许多可通过激活Nrf2来治疗阿尔茨海默病的药物，如白藜芦醇、吡哆醇、正丁基苯酞等，也已进入临床试验。此外靶向Nrf2在心血管疾病的治疗中具有广阔的前景。SIRT1（Sirtuin 1）是一种NAD依赖性脱乙酰酶，在糖尿病引起的多种心血管疾病中发挥重要作用。在糖尿病Sprague道利大鼠模型中，可增加Nrf2在细胞核中的定位，从而减轻心肌缺血-再灌注损伤^[67]^。并且铁抑素已被证明可防止脑、肝、肾和心脏中的缺血性损伤，并在许多细胞和动物模型中减少神经退行性表型，以及防止心肌细胞中过量铁诱导的铁死亡^[59,68,69]^。利用Nrf2信号转导途径对铁死亡进行激活或抑制的药理学调节已被证明是有效的，这取决于疾病背景。

## 6 总结与展望

随着对铁死亡的深入研究，许多关键的抗铁死亡成员分子都被发现受到Nrf2的转录调控。因此Nrf2在铁死亡调控中的重要性日益凸显。Nrf2作为细胞抗氧化反应的关键调节因子，广泛参与铁、脂质、氨基酸等代谢。因此，通过Nrf2及其下游效应物的药理学调节，可以实现诸如逆转癌症耐药，预防和治疗多种疾病包括神经退行性疾病、心血管疾病等。因此靶向Nrf2信号通路的治疗策略可用于多种铁死亡相关疾病，具有广阔的治疗前景。
